# Perceptions and Misconceptions in Molecular Recognition: Key Factors in Self-Assembling Multivalent (SAMul) Ligands/Polyanions Selectivity

**DOI:** 10.3390/molecules25041003

**Published:** 2020-02-24

**Authors:** Domenico Marson, Erik Laurini, Suzana Aulic, Maurizio Fermeglia, Sabrina Pricl

**Affiliations:** 1 Molecular Biology and Nanotechnology Laboratory (MolBNL@UniTS), Department of Engineering and Architecture, University of Trieste, 34127 Trieste, Italy; domenico.marson@dia.units.it (D.M.); saulic@units.it (S.A.); maurizio.fermeglia@units.it (M.F.); sabrina.pricl@dia.units.it (S.P.); 2 Department of General Biophysics, Faculty of Biology and Environmental Protection, University of Lodz, 90-236 Lodz, Poland

**Keywords:** self-assembly, multivalency, amphiphilic ligands, DNA, heparin, polyanion binding, isothermal titration calorimetry, molecular simulations, chirality

## Abstract

Biology is dominated by polyanions (cell membranes, nucleic acids, and polysaccharides just to name a few), and achieving selective recognition between biological polyanions and synthetic systems currently constitutes a major challenge in many biomedical applications, nanovectors-assisted gene delivery being a prime example. This review work summarizes some of our recent efforts in this field; in particular, by using a combined experimental/computation approach, we investigated in detail some critical aspects in self-assembled nanomicelles and two major polyanions—DNA and heparin.

## 1. Introduction

Biological systems efficiently and spontaneously exploit the phenomenon of molecular recognition at a nanointerface to organize the self-assembly of ligands for binding to different biomolecular targets [1,2]. Aside from natural compounds, many synthetic supramolecular systems are endowed with the capacity of binding biological targets at the nanoscale level, those ligands exploiting multivalent binding features being particularly effective [3]. In this context, multivalency refers to the concurrent interaction of several binding moieties on a single (supra)molecular object with the matching binding sites on another (supra)molecular entity. Typically, this strategy is widely employed by biological systems to achieve high-affinity binding in challenging environments, e.g., physiological solutions or blood [4]. Self-assembling nanotechnology is a powerful strategy to organize such interactions [5], and in our previous work we introduced the concept of self-assembled multivalent (SAMul) approach to define those situations in which auto-aggregated supramolecular entities precisely displaying specific ligands on their surface are able to establish multivalent interactions with their targets [6,7,8,9,10,11,12,13,14,15,16,17].

At the same time, life is dominated by polyanions, including cell membranes, nucleic acids, microfilaments and tubules, and polysaccharides [18], and while biology can govern these anionic molecules with specific selectivity, human understanding and mastering of this “polyanion world” still constitutes a major scientific hurdle. DNA and heparin are two prototypes of biological polyanions characterized by a high negative charge density, which have attracted enormous biomedical interest in many disparate fields of application, including—but by no means limited to—gene delivery (for DNA) [19] and coagulation control in major surgical operation (for heparin) [20]. Accordingly, the scientific community has focused major efforts in discovering or synthesizing a plethora of molecular entities able to selectively binding these two biomacromolecules [21,22]. However, binding selectivity by different cationic ligands with respect to these two primary polyanions has rarely been explored, the main reason for this likely residing in the fact that DNA and heparin typically locate in different biological compartments; accordingly, they seldom compete for the same ligands. Nonetheless, there are important exceptions to this, as for instance in bacterial biofilms (which are populated by extracellular DNA that competes with heparin for binding to the same proteins [23]), and in gene-based therapeutics, where delivery vectors must shuttle the genetic material (DNA, RNA, miRNA or oligonucleotides) in the blood stream and through the cellular membrane, where heparin and other glycosaminoglycans can be encountered.

Obviously, if we could identify the main aspects driving selective polyanion binding by natural and/or human-designed cationic ligands, we would also be able to produce chemical systems that could be substantially more efficient and effective in biomedical processes and be better tailored for definite clinical applications. In this respect, during the last years we have reported systems in which the SAMul concept was used to display specific cationic amphiphilic ligands at DNA/RNA/heparin binding nanointerfaces [6,7,8,10,11,12,13,14,15,16,17,24,25,26,27]. There are intrinsic benefits of this approach such as simple synthetic pathways, ligand chemical variety, programmable morphology, and the facility to disassemble the nanostructures in response to chemical/physiological stimuli. Moreover, in recent efforts we have focused on investigating if and why DNA and heparin might be endowed with differential binding preferences towards a variety of SAMul nanoassemblies, proving that factors such as ligand chemical structures and chiral features have a profound impact on polyanion affinities, irrespectively of the charge density of the SAMul micelles. In all these systems, the SAMul ligands reside at the binding interface and play a key role in optimizing the interactions between the corresponding nanomicelles and the polyanions, suggesting a molecular mechanism at the nanoscale for these outcomes. And is exactly the description of these effects, coupled with thermodynamic and structural insights into polyanion recognition by nanomicelles originated by the self-assembly of different amphiphilic ligands, and their relevant practical implications which are the focus of the present review, as it develops below.

## 2. Thermodynamic Insight into SAMul-Driven Polyanion Recognition

### 2.1. Effect of SAMul Ligands Surface Groups in Polyanion Recognition

**Figure 1:**
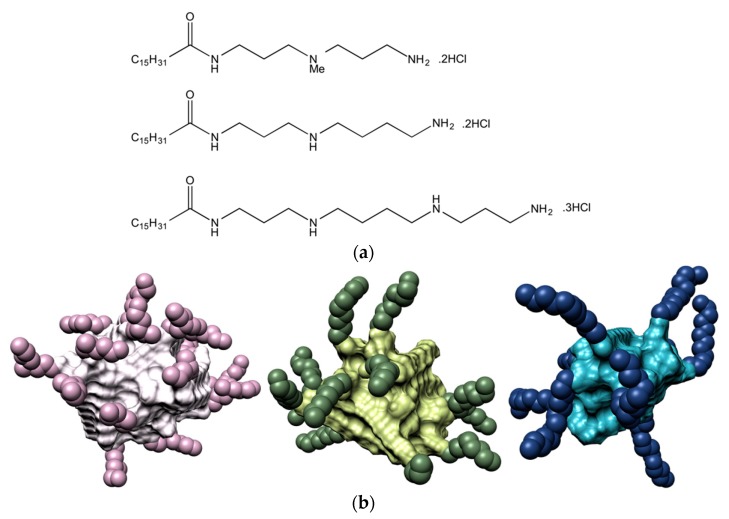
(**a**) Chemical structure of the three SAMul ligands C_16_-DAPMA (top), C_16_-SPD (center), and C_16_-SPM (bottom); (**b**) Molecular models, as extracted from equilibrate atomistic molecular dynamics (MD) simulation of the micelles formed upon self-assembling of the C_16_-DAPMA (left), C_16_-SPD (middle), and C_16_-SPM (left) amphiphilic ligands. The C_16_ hydrophobic portion in each micelle is shown as while, light green and light blue spheres, while the corresponding DAPMA, SPD and SPM residues are portrayed as lavender, dark green, and navy-blue spheres, respectively. Water molecules, ions and counterions are not shown for clarity.

For the purpose of investigating the effect of the ligand head groups on polyanion recognition by SAMul ligands, we considered spermine (SPM), spermidine (SPD) and the shorter *N*,*N*-di-(3-aminopropyl)-*N*-methylamine (DAPMA) as the polyanion-binding ligands [13]. Mammalian cells naturally produce spermine and spermidine (along with their precursor putrescine), which play important roles in many cellular processes including regulation of transcription and translation, control of ion channels activity, modulation of kinase activities, effects on the cell cycle, protection from oxidative damage and maintenance of membrane structure/function. Above all, these two polyamines are known to physiologically interact with DNA in diverse and important ways, functioning as a protective agent, and a modulator of the nucleic acid secondary structure [28]. In order to create the corresponding amphiphilic, self-assembling ligands, we initially selected palmitic acid (C_16_) as the hydrophobic moiety, and connected the hydrophobe to the different amines via 2-(1H-benzotriazole-1-yl)-1,1,3,3-tetramethylaminium tetrafluoroborate (TBTU)-mediated peptide coupling with an appropriate group strategy [13]. The synthesis yielded the three C_16_-DAPMA, C_16_-SPD, and C_16_-SPM ligands, with nominal charge at pH = 7.4 of +2, +2, and +3, respectively (Figure 1a). In aqueous solution at physiological pH and ionic strength (7.4, 150 mM NaCl), all three SAMul ligands were able self-assemble into almost spherical nanomicelles (Figure 1b), whose main characteristics are listed in Table 1.

The three different SAMul nanomicelles were next challenged for DNA and heparin binding. Experimentally, DNA binding was investigated via the ethidium bromide (EthBr) assay, during which the displacement of EthBr from DNA by each SAMul micelle is monitored by fluorimetry. Heparin binding was quantified via a heparin binding competition assay (HBCA) exploiting Mallard Blue (MB). MB is a highly charged (+5), blue-colored synthetic dye based on an arginine-functionalized thionine developed by our group as a specific heparin sensor. MB works in both physiological solutions and serum [6,7,8,10,11,12,29,30,31,32], and reports on heparin binding by UV−visible spectroscopy. Both displacement assays allow for the rapid determination of two binding parameters: CE_50_ (i.e., the cation:anion charge excess at which 50% of each indicator dye is displaced from its complex) and EC_50_ (i.e., the effective concentration at which 50% of each dye is displaced). Accordingly, the lower the values of CE_50_ and EC_50_ obtained wither in the MB or EthBr displacement assay, the stronger the affinity of the tested binder with respect to heparin and DNA, respectively.

In parallel, binding of the three nanomicelles to the two polyanions was investigated again experimentally - by Isothermal Titration Calorimetry (ITC)—and in silico, using atomistic molecular dynamics (MD) simulations. These two, more sophisticated techniques allow for the obtainment of the full thermodynamic characterization of each nanomicelle/polyanion interaction, i.e., the binding enthalpy ∆H_bind_, binding entropy T∆S_bind_, and free energy of binding ∆G_bind_.

**Figure 2:**
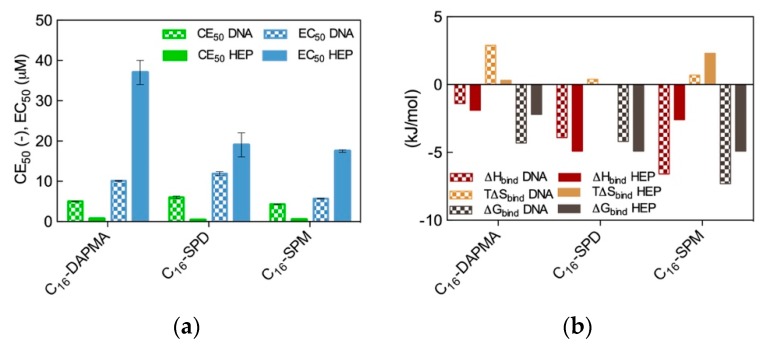
(**a**) DNA and heparin binding parameters CE_50_ and EC_50_ for the three SAMul micelles as obtained from EthBr and MB displacement assays; (**b**) Thermodynamics parameters for the binding of the three SAMul micelles to DNA and heparin as determined by ITC. Adapted from [13], published by RSC, 2016.

The results from the two spectrometric assays are shown in Figure 2a, while the corresponding binding thermodynamics data are reported in Figure 2b.

As seen from Figure 2a, the preliminary results based on the dye displacement assays revealed that the micelles formed by the amphiphilic ligands featuring the most charged ligands (C_16_-SPM) were optimized for DNA binding (CE_50_ = 4.3), followed by the C_16_-DAPMA (5.0) and the C_16_-SPD (6.0) nanoassemblies, respectively. On the contrary, the same analysis showed that the nanomicelle/heparin binding charge-efficiency decreased in the order C_16_-SPD (CE_50_ = 0.34) > C_16_-SPM (0.49) > C_16_-DAPMA (0.69). Although these differences in CE_50_ values might seem rather small, they were statistically meaningful and reproducible; as such, they provided a first clue on the fact that DNA and heparin can behave differently when facing these SAMul nanostructures as binding partners, even in the case when the nominal charge on the hydrophilic portion of the amphiphilic ligands was the same (e.g., +2 for both DAPMA and SPD).

We were somewhat taken aback by the apparent DNA/heparin selectivity difference discussed above as we had originally anticipated that both polyanion would bind the most highly charged micelles best or, at least, all three SAMul nanoobjects equally well on the basis of their predicted micellar surface density values (σ_mic_ = 0.026, 0.024, and 0.026 e/nm^2^ for C_16_-DAPMA, C_16_-SPD, and C_16_-SPM, respectively, Table 1). Thus, we decided to verify this polyanion binding selectivity via a direct method (ITC). A comparison of the ITC-derived binding thermodynamics parameters (Figure 2b) with the trends obtained from the displacement assays (Figure 2a) revealed the broad agreement between the two data sets. Indeed, the micelles generated by the C_16_-SPM SAMul ligands were again found to be the most effective DNA binders (∆G_bind_ = −7.3 kJ/mol), while the other two SAMul nanomicelles presented comparable affinity for the nucleic acid (∆G_bind_ = −4.3 kJ/mol and −4.2 kJ/mol for C_16_-DAPMA and C_16_-SPD, respectively). At the same time, ITC data consistently ranked the C_16_-SPD micelles as the best heparin binders together with C_16_-SPM (∆G_bind_ = −4.9 kJ/mol for both systems) and C_16_-DAPMA as the SAMul nanostructures with lowest affinity towards this anionic polysaccharide (∆G_bind_ = −2.2 kJ/mol). When considering the per-charge-normalized binding free energy values (∆G_bind_^+^), ITC data completely matched the dye displacement trends, particularly for heparin for which we obtained ∆G_bind_^+^ = −2.45 kJ/mol for C_16_-SPD, −1.63 kJ/mol for C_16_-SPM, and −1.1 kJ/mol for C_16_-DAPMA, respectively.

**Figure 3:**
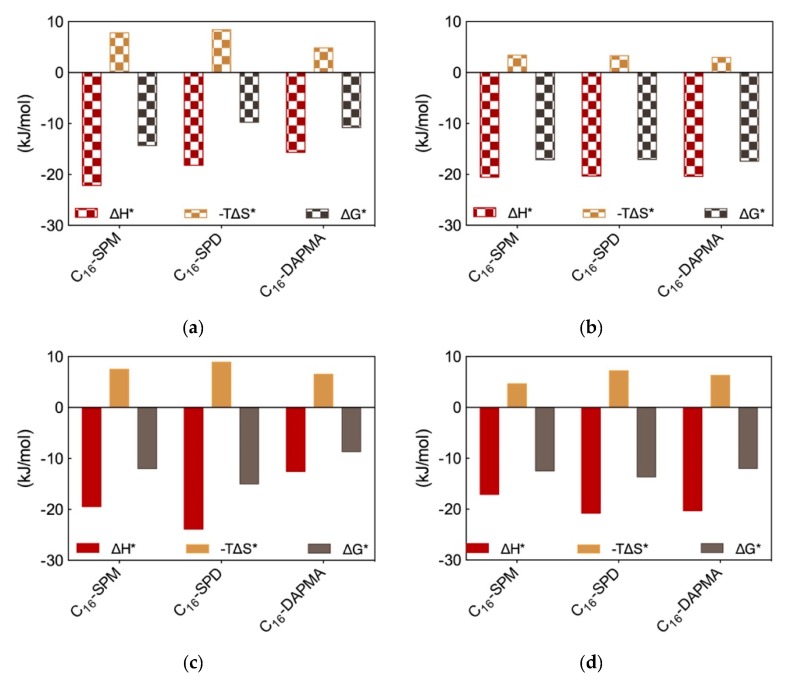
Charge-normalized per-residue effective free energy of binding ∆G*, enthalpy (∆H*), and entropy (-T∆S*) for (**a**) each SAMul micelle in complex with DNA; (**b**) DNA bases in complex with each SAMul micelle; (**c**) each SAMul micelle in complex with heparin; (**d**) heparin sugars in complex with each SAMul micelle. See text for explanations. Adapted from [13], published by RSC, 2016.

Having ascertained by transmission electron microscopy (TEM) that all cationic micelles remained intact upon polyanion binding, and that they were organized into hierarchical nanostructure arrays resulting in close packed interactions with both polyanions, we finally resorted again to atomistic MD simulations in order to provide an explanation for the displacement assays/ITC data and to get some insights into the binding interface between the three SAMul nanostructures and the negatively charged macromolecules. These results are shown in Figure 3.

The MD trajectory analysis for each DNA/SAMul micelle complex performed from the viewpoint of the self-assembled ligands allowed us to observe that, during binding, the micelles formed by C_16_-SPM contacted the nucleic acid with 9 out of 10 available SAMuls (i.e., the so-called effective residues), resulting in a favorable charge-normalized, per-effective-residue enthalpic gain ∆H*. This overcompensated the corresponding entropic cost (T∆S*) associated with ligand organization upon polyanion binding, leading to a favorable free energy of binding ∆G* of −14.32 kJ/mol (Figure 3a), in agreement with ITC data (Figure 2b). The other two micelles formed by the less-charged ligands (2+) both gained less in ∆H* (Figure 3a), since the C_16_-DAPMA and C_16_-SPD nanostructures only used 7 and 8 effective residues (out of 16 and 13 available residues, respectively) to bind DNA. Yet, the micelles composed by the shorter, slightly more rigid ligand C_16_-DAPMA payed less entropic penalty upon DNA interaction, and this slightly favored their nucleic acid affinity (∆G* = −10.80 kJ/mol) over C_16_-SPD micelles (∆G* = −9.76 kJ/mol) (Figure 3a), in line with the experimental ITC results (Figure 2b). In aggregate, the simulated ∆G* values followed the same trend as the experimental CE_50_ (Figure 2a) and ITC values (Figure 2b), with the DNA affinity of these SAMul micelles decreasing in the order C_16_-SPM < C_16_-DAPMA > C_16_-SPD.

Applying the same analysis this time from the perspective of each anionic DNA residue implicated at the binding interface, the values of the three charge-normalized, per-effective-residue thermodynamic parameters ∆G*, ∆H* and T∆S* were essentially independent of the ligand—from the viewpoint of DNA, all interface interactions were equally good (Figure 3b). This result led us to conclude that the selectivity of the SAMul micelles towards DNA can be ascribed only to ligand optimization—as such DNA appears to be a shape persistent polyanion which simply binds to, and organize the SAMul display with which it is presented.

For heparin binding, starting again from the perspective of the SAMul effective charge, the C_16_-SPD micelles experienced the largest ∆H* gain upon reorganizing and optimizing the highest number of effective residues (12/13) while interacting with the polysaccharide with respect to the micelles formed by their other two counterparts C_16_-DAPMA (9/16) and C_16_-SPM (6/10) (Figure 3c)—in line with the ITC data (Figure 2b). Although the entropic loss for the C_16_-SPD/heparin system was not the best in the series, the overall binding remained enthalpy-driven in nature, confirming the heparin best-binding properties of this SAMul nanosystem (∆G* = −14.98 kJ/mol) over the C_16_-SPM (−11.97 kJ/mol) and C_16_-DAPMA (−8.65 kJ/mol) ones (Figure 3c), in keeping with both CE_50_ (Figure 2a) and ITC data (Figure 2b). Considering SAMul/heparin binding from the perspective of each heparin sugar (Figure 3d), a different behavior could also be observed, depending on the ligand nature: each heparin residue offset the entropic cost of binding C_16_-SPD micelles with a greater enthalpic gain of its own. This was in stark contrast to DNA where, as discussed above, each anion behaved identically irrespective of the ligand. As such, the C_16_-SPD nanomicelles induced more effective binding from each residue of the heparin chain via an enthalpy/entropy optimization, mediated through polyanion structural adaptation—i.e., heparin can be defined as an adaptive polyanion, which not only binds to the SAMul display, but importantly, is also able to adapt itself in response.

Thus, this part of our work highlighted the first misconception in ligand choice for generating selective SAMul displays at polyanion binding interfaces according to which polyanion and ligand charge density are the unique main players in driving electrostatic ion–ion binding—and introduced the two new concepts of ligand structural details and polyanion adaptability at binding interfaces as the two key parameters required for productive polyanion selectivity—as confirmed by the complementary experimental methods of competition binding assays, ITC and molecular simulations.

### 2.2. Effect of SAMul Hydrophobic Tails in Polyanion Recognition

**Figure 4:**
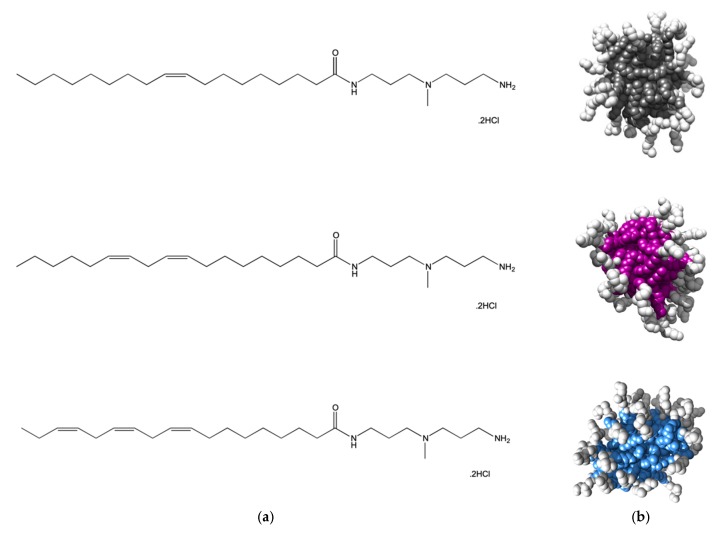
(**a**) Structures of the amphiphilic C_18_-DAPMA ligands bearing 1 (C_18,1_-DAPMA, top), 2 (C_18,2_-DAPMA, middle), and 3 (C_18,3_-DAPMA, bottom) *cis*-alkene groups, respectively. (**b**) Molecular models, as extracted from equilibrate atomistic MD simulation of the micelles formed upon self-assembling of the C_18,1_-DAPMA (top), C_18,2_-SPD (middle), and C_18,3_-SPM (bottom) amphiphilic ligands. Each hydrophobic micellar core is shown as colored spheres while the hydrophilic DAPMA portion is portrayed as white spheres. Water molecules, ions and counterions are omitted for clarity.

Although the hydrophobic moieties of amphiphilic molecules are responsible for driving self-assembly in aqueous environments, once the micellization process is completed they locate inside the resulting nanostructures and, as such, should not directly interfere with polyanion binding. Nonetheless, we decided to challenge the validity of this general concept and, since the self-assembled micelles of all three SAMul ligands C_16_-DAPMA, C_16_-SPD, and C_16_-SPM were excellent systems for polyanion binding selectivity, we next decided to use closely-related ligands to explore the eventual role played by the hydrocarbon tails on their self-assembling properties and the related DNA/heparin binding [12]. To the scope, we prepared three new molecular entities still featuring DAPMA as the polar head yet this time decorated with C_18_ aliphatic chains bearing 1, 2 or 3 double bonds, respectively (see Figure 4a). The reason underlying the choice of the C_18_ instead of the C_16_ fragment adopted in the study discussed above was solely due to the possibility of incorporating up to 3 double bonds in the hydrophobic portion while maintaining the identical and flexible 7-carbon-long segment within the same moiety.

Atomistic MD simulations initially predicted all compounds in Figure 4 to self-assembly into spherical micelles of nanometric dimensions (Figure 4b). For this nanomicelle set, the average micellar diameters (D_mic_), aggregation number (N_agg_) and zeta potential (ζ) values were computationally predicted to increase in passing from one to two C=C bonds in the alkyl chain while the presence of a further unsaturation resulted in a relatively confined additional effect (Table 2). The subsequent experimental characterization of the relevant self-assembled forms fully confirmed the in silico data, as also shown in Table 2.

**Figure 5:**
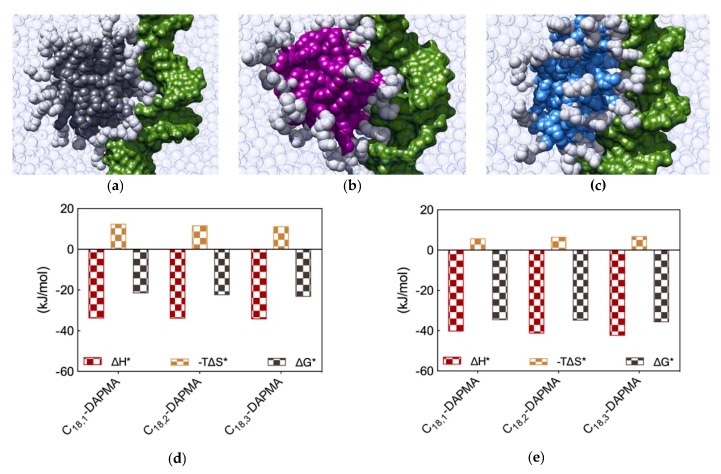

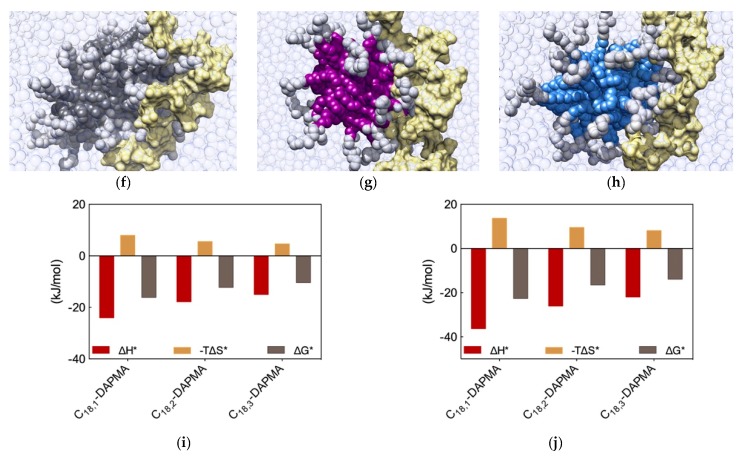
Equilibrated atomistic MD simulation snapshots for DNA in complex with (**a**) C_18,1_-DAPMA, (**b**) C_18,2_-DAPMA and (**c**) C_18,3_-DAPMA micelles. (**d**) Charge-normalized per-residue effective free energy of binding ∆G*, enthalpy (∆H*), and entropy (−T∆S*) for each SAMul micelle in complex with DNA and (**e**) DNA bases in complex with each SAMul micelle. Equilibrated atomistic MD simulation snapshots for heparin in complex with (**f**) C_18,1_-DAPMA, (**g**) C_18,2_-DAPMA and (**h**) C_18,3_-DAPMA micelles. (**i**) Charge-normalized per-residue effective free energy of binding ∆G*, enthalpy (∆H*), and entropy (−T∆S*) for each SAMul micelle in complex with heparin and (**j**) heparin sugars in complex with each SAMul micelle. See text for explanations. Adapted from [12], published by RSC, 2017.

Binding of the two polyanions with the nanomicelles formed by the three different SAMuls was next studied using again a combination of EthBr/MB displacement assays and MD simulations. Data the experimental techniques demonstrated that, for DNA, the SAMul micelle binding affinity decreased in the order C_18,3_-DAPMA > C_18,2_-DAPMA > C_18,1_-DAPMA (CE_50_ = 3.5, 4.3 and 5.0, respectively) while, for heparin, the opposite behavior was observed with polyanion binding ability decreasing with decreasing unsaturation level, i.e., C_18,1_-DAPMA > C_18,2_-DAPMA > C_18,3_-DAPMA (CE_50_ = 0.80, 1.8, ad 2.3, respectively) [12]. Given the fact that the SAMul ligand head is the same for the three amphiphiles, and that the molecular-scale structural differences of these SAMul systems are buried in respective micellar core, this apparent polyanion selectivity was quite surprising. Thus, MD simulations were finally performed to explain this somewhat unanticipated findings (Figure 5a–c,f–h). A first, qualitative analysis of the MD data revealed that the micelles formed by C_18,1_-DAPMA could exploit 19 effective residues (out of 28) in stably binding heparin whereas a progressive decreased in the number of effective residues was estimated when considering C_18,2_-DAPMA (15/32) and C_18,3_-DAPMA (13/35) in complex with the anionic polysaccharide, respectively. When bound to DNA, however, this difference in effective residues for each micelle type leveled off, being equal to 16, 17, and 18 for ligands bearing 1, 2, and 3 unsaturation in their aliphatic portion, respectively.

Further analysis of each SAMul/polyanion complex performed as detailed in Section 2.1 indeed revealed the molecular reasons for these findings. Starting the discussion with heparin, and again considering polyanion binding from the viewpoint of each single effective SAMul interaction (Figure 5d), the more flexible nature of the mono-unsaturated chain of C_18,1_-DAPMA allow this SAMul micelles to maximize their interactions with the polysaccharide, resulting in the most favorable enthalpic contribution (∆H* = −24.02 kJ/mol), which overcompensate the unfavorable penalty paid upon binding (T∆S* = −7.92 kJ/mol) and ultimately leading to the largely favorable ∆G* value of −16.10 kJ/mol (Figure 5d). As discussed a few lines above, increasing the rigidity of the hydrocarbon chain has the effect of decreasing the number of micelle effective residues involved in heparin productive binding, and this reflects into a progressive decrement of the corresponding binding enthalpic component (∆H* = −17.76 and −14.98 kJ/mol for C_18,2_-DAPMA and C_18,3_-DAPMA, respectively). However, the inclusion of 2 or 3 double carbon-carbon bonds in the hydrophobic moieties of the SAMul ligands has a beneficial effect on the corresponding binding entropy (T∆S* = −5.55 and −4.63 kJ/mol for C_18,2_-DAPMA and C_18,3_-DAPMA, respectively) since, as these molecules become progressively more rigid, they are less subjected to conformational reorganization upon binding and, as such, also suffer less entropic penalty. As a net result, the heparin binding of these two SAMul micelles however remain enthalpically driven (the corresponding ∆G* being equal to −12.21 and −10.35 kJ/mol for C_18,2_-DAPMA and C_18,3_-DAPMA, respectively).

Overall, the affinity of the three SAMul micelles for heparin, as expressed by the corresponding simulated values of ∆G*, indeed follow the same trend as the corresponding CE_50_ values, that is C_18,1_-DAPMA > C_18,2_-DAPMA > C_18,3_-DAPMA.

When considering DNA binding, simulation results highlight an interesting feature: the enthalpic gain for the three different SAMul micelles upon interaction with the nucleic acid is almost constant and equal to ∆H* = −33.77, −33.81, and −34.15 kJ/mol for C_18,1_-DAPMA, C_18,2_-DAPMA, and C_18,3_-DAPMA, respectively (Figure 5e). Thus, in absolute terms, the small difference in effective binding enthalpy between the micelles formed by the least and the most unsaturated SAMul micelle (0.38 kJ/mol) is in stark contrast with what observed in the case of heparin binding, where the same difference amounts to 9.04 kJ/mol (Figure 5d). Also, the entropic component is decidedly less subjected to variation when these three micelles are placed in contact with DNA, the difference between C_18,1_-DAPMA and C_18,3_-DAPMA being again limited to 1.3 kJ/mol (Figure 5e) while, for heparin, it is almost this value is almost the double (3.3 kJ/mol, Figure 5d). As a result, the values of DG* for the DNA binding by these three SAMul micelles are definitely less sensitive to each SAMul molecular structure and, in agreement with their corresponding experimental CE_50_ values, they rank the affinity of the micelles for the polyanion in the order C_18,3_-DAPMA > C_18,2_-DAPMA > C_18,1_-DAPMA (∆G* = −23.07, −22.27, and −21.43 kJ/mol, respectively, Figure 5e).

The same analysis performed on the heparin perspective showed that both the heparin sugars and the micellar ligands experienced the same trend in the variation of the three binding components (Figure 5i). Accordingly, upon binding the micelles generated by the most flexible ligand (C_18,1_-DAPMA) the polyanion can cope with a higher entropy loss via a considerably more favorable enthalpic stabilization. At the same time, this polyanion is able to gradually adjust the enthalpy/entropy compensation as the micellar rigidity increases, a phenomenon utterly analogous to that observed when studying the effect of the ligand head effect on polyanion selectivity discussed in Section 2.1. Once again, these data confirm the concept that heparin is a relatively flexible adaptive polyanion. From the DNA viewpoint, the values of ∆G* and its components ∆H* and T∆S* are aging largely independent of the hydrophobic portion of the SAMul ligands (Figure 5j), confirming that each DNA units binds its cationic counterpart with comparable strength. In agreement with what presented in Section 2.1, DNA confirms to be a relatively rigid and shape-persistent polyanion.

Thus, the new concept we learned from this study is that, contrarily to an intuitive misconception, even though the molecular structural differences in these new three ligands are buried in the hydrophobic cores of the micelles they generate, with a mechanisms prototypical of SAMul materials these characteristics are transmitted through the entire nanoobjects, ultimately resulting into significantly different polyanion binding preferences, with heparin—an adaptive polyanion—being more affine to the micelles constituted by the most flexible monomers.

### 2.3. Effect of SAMul Chirality in Polyanion Recognition

Chiral recognition by DNA and heparin is not a new concept. For instance, the chiral discriminating capabilities of heparin have been exploited in capillary electrophoresis [33] for the separation of several chiral drugs [34]. Contextually, DNA is an inherently chiral molecule, as its constituents contain several stereogenic centers. As a matter of fact, DNA global handedness is responsible for the nucleic acid recognition by chiral molecules and, indeed, DNA has been exploited as a very efficient chiral stationary phase [35] and as a chiral microenvironment or chiral template in asymmetric synthesis [36].

**Figure 6:**
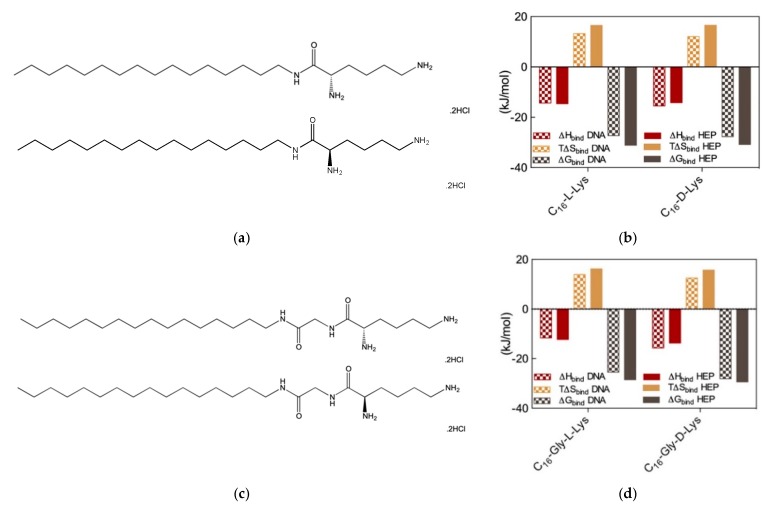
(**a**) Structures of the amphiphilic C_16_-l-Lys (top) and C_16_-d-Lys (bottom) ligands. (**b**) Thermodynamics parameters for the binding of the micelles formed by the two chiral SAMuls in (**a**) to DNA and heparin as determined by ITC. (**c**). Structures of the amphiphilic C_16_-Gly-l-Lys (top) and C_16_-Gly-d-Lys (bottom) ligands. (**d**) Thermodynamics parameters for the binding of the micelles formed by the two chiral SAMuls in (**b**) to DNA and heparin as determined by ITC. Adapted from [14], published by RSC, 2016.

Therefore, we wondered whether chiral SAMul systems could achieve enantioselectivity in the two polyanion binding. To the purpose, we initially synthesized two new amphiphilic cationic ligands C_16_-l-Lys and C_16_-d-Lys (Figure 6a), which are utterly identical apart from the chirality of the lysine ligands [14]. As expected, these two chiral SAMul molecules self-assembled – at the same CMC (45 and 48 μM for the l and d SAMul, respectively)—into spherical nanomicelles with similar dimensions (D_mic_ = 6.2 ± 1.7 and 6.3 ± 1.7 nm) and surface charges (ζ = + 45.2 ± 1.6 and 39.2 ± 1.6 mV) but opposed chirality. It was therefore not surprising to find that these two nanostructures failed to show any preference in binding heparin and DNA, the corresponding CE_50_ values being equal to 1.8 ± 0.1 and 1.8 ± 0.1 for heparin binding and to 1.6 ± 0.2 and 1.7 ± 0.1 for DNA binding by C_16_-l-Lys and C_16_-d-Lys, respectively. The results from MB and EthBr displacement assays were definitively confirmed by ITC (Figure 6b), which confirmed the absence of chiral recognition in the polyanion binding thermodynamics by the two SAMul nanomicelles (e.g., for DNA ∆G_bind_ = −27.3 and −27.7 kK/mol and for heparin ∆G_bind_ = −31.1 and −30.8 kJ/mol for C_16_-l-Lys and C_16_-d-Lys, respectively).

Although these results suggested that self-assembled nanoscale chirality has no significant impact on the molecular recognition interfaces, we were still not satisfied and went on by synthesizing two closely related molecules, C_16_-Gly-l-Lys and C_16_-Gly-d-Lys, featuring a glycine spacer between the C_16_ hydrophobic chain and the L/D lysine polar head of the ligand (Figure 6c). The self-assembly of these two new chiral SAMuls also resulted in small and spherical micelles with similar characteristics (e.g., CMC = 49 and 49 μM and ζ = 40.1 ± 0.1 and 47.1 ± 0.1 mV for C_16_-Gly-l-Lys and C_16_-Gly-d-Lys, respectively). Pleasingly, however, the results from the two, preliminary dye-based assays revealed that chiral discrimination at the nanomicelle-polyanion binding interface has been switched on by the presence of the glycine spacer unit. In fact, the EthBr displacement assay indicated that the DNA binding ability of C_16_-Gly-l-Lys and C_16_-Gly-d-Lys was significantly different, the corresponding CE_50_ values being equal to 3.8 ± 0.7 and 1.5 ± 0.1, respectively. At the same time, also with respect to heparin the two new chiral SAMuls displayed different performances, with CE_50_ values of 1.7 ± 0.2 and 1.1 ± 0.1 or C_16_-Gly-l-Lys and C_16_-Gly-d-Lys, respectively. The chiral discrimination of the two polyanions by these SAMul nanostructures was again confirmed by ITC experiments (Figure 6d). The relevant binding thermodynamic parameters indeed showed that DNA displays a clear preference for the micelles formed by the SAMul D-enantiomer over those generated by the amphiphilic ligand with opposed chirality, with ∆G_bind_ values of −28.1 and −25.5 kJ/mol for C_16_-Gly-d-Lys and C_16_-Gly-l-Lys, respectively. Furthermore, the same techniques indicated that also heparin binds the D-SAMul micelles with a slight preference (∆G_bind_ = −29.4 kJ/mol) over the L-SAMul ones (∆G_bind_ = −28.5 kJ/mol). A more detailed analysis of the polyanion binding thermodynamics by these two chiral SAMul nanosystems showed that the binding enthalpy is always negative (i.e., exothermic binding), as it could be expected from interaction driven by electrostatic forces. The ∆H_bind_ values for DNA binding are −15.7 and −11.6 kJ/mol while those for heparin binding are −13.7 and 12.3 kJ/mol for C_16_-Gly-d-Lys and C_16_-Gly-l-Lys, respectively. The binding entropies are also favorable (i.e., positive, Figure 6), suggesting the combined effect of some stabilizing hydrophobic interactions between the CH_2_-group of the micelle terminal Lys moieties and the bases/sugars of the polyanions and an increment in the degree of disorder of the overall system induced by the release of water molecules and counterions upon nanoscale binding interface formation. Interestingly, however, the entropic differences between the enantiomeric micelles are less pronounced, as for DNA the T∆S_bind_ values are equal to +15.7 and +16.2 kJ/mol and for heparin these quantities amount to +12.5 and +13.9 kJ/mol for C_16_-Gly-d-Lys and C_16_-Gly-l-Lys, respectively. In aggregate, these results clearly show that the enhanced DNA binding—and, albeit to a lesser extent, heparin binding—of the C_16_-Gly-d-Lys-based nanomicelles is an enthalpically-driven process. As such, the specific SAMul-polyanion recognition appears to be optimized on the surface of the C_16_-Gly-d-Lys micelles in comparison with the otherwise identical nanoassemblies formed by the C_16_-Gly-l-Lys amphiphilic ligands. We also reasoned that the lower degree of heparin recognition exhibited by these two micelles could be the results of the greater heparin polydispersity, which ultimately reflects into a less-well defined distribution of the anionic sites along the polysaccharide backbone. DNA, as a more rigid and less-adaptive polyanion (Section 2.1), is able to present its anionic sited evenly and more regularly spaced down its helical structure, thereby likely benefitting more from a suitably structured binding counterpart.

In summary, the further new concept we learned from this study is that, contrarily to another intuitive misconception, chiral discrimination and differential polyanion recognition by otherwise identical nanomicelles can be switched on by the presence of specific molecular features—such as an apt spacer connecting the hydrophobic and hydrophilic portions of the micellar amphiphilic constituents—which ultimately results in the differential optimization of the relevant binding nanointerfaces. Understanding such effects in details can add important criteria for the design of new SAMul ligands with enhanced chiral recognition to be generally exploited in optimizing binding process at self-assembled bio-interfaces (e.g., cell membranes, proteins and other polyanions).

### 2.4. Effect of SAMul Chirality in Polyanion Recognition—Revisited

**Figure 7:**
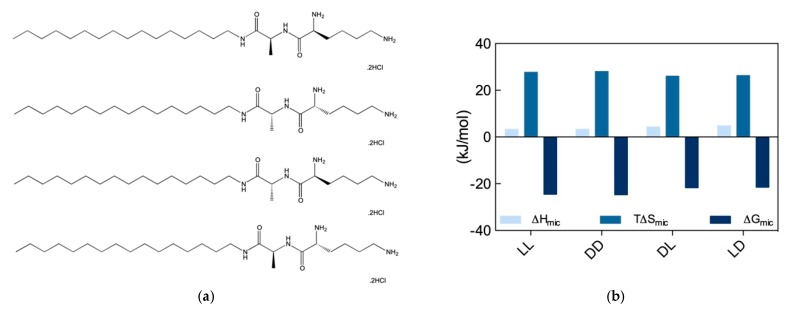
(**a**) Structures of the amphiphilic chiral ligands (from top to bottom) C_16_-l-Ala-l-Lys, C_16_-d-Ala-d-Lys, C_16_-d-Ala-l-Lys, and C_16_-l-Ala-d-Lys. (**b**) Thermodynamics parameters for micellization of the chiral SAMuls in (**a**) as determined by ITC. Adapted from [8], published by John Wiley & Sons, 2018.

Given the novelty and the practical application potential of the results discussed in Section 2.3, we decided to gain a deeper understanding of the recognition potential of chiral SAMul systems towards the two prototypical biological polyanions, DNA and heparin. To the purpose, another set of stereoisomeric SAMul amphiphiles were designed and synthesized, as shown in Figure 7a. By virtue of the presence of the two amino acids Ala and Lys, each of these molecules contains two chiral centers; accordingly, they can exist in four possible stereoisomers, i.e., two enantiomer pairs with a diastereomeric relationship to each other, as follows: C_16_-l-Ala-l-Lys and C_16_-d-Ala-d-Lys (ll and dd), and C_16_-d-Ala-l-Lys and C_16_-l-Ala-d-lys (dl and ld), respectively [8]. We reasoned that, in contrast to our previous work discussed above, the presence of a second chiral center (Ala) that is not directly involved in the binding interface, should allow us to explore both enantiomeric and diastereomeric effects on polyanion binding selectivity.

The self-assembly of these four new SAMul ligands was initially explored by ITC, according to which the ll and dd nanosystems were characterized by a substantially lower CMC (52 and 48 μM for the ll and dd, respectively) than the dl and ld ones (159 and 172 μM, respectively). Interesting, however, at variance with what observed in our previous work (Section 2.3), for these new amphiphiles the micellization process was entropically driven (T∆S_mic_ > 0) and slightly enthalpically disfavored (∆H_mic_ > 0) (Figure 7b). In particular, the analysis of the micellization thermodynamic data reported in Figure 7b reveals that micelle formation by the ll and dd SAMuls are both slightly enthalpically and entropically preferred over the micellization of the diastereomeric dl and ld SAMuls. Accordingly, the relevant ∆G_mic_ values becomes more favorable, in the order: ll (∆G_mic_ = −24.5 kJ/mol) ≈ dd (∆G_mic_ = −24.7 kJ/mol) > dl (∆G_mic_ = −21.7 kJ/mol) ≈ ld (∆G_mic_ = −21.5 kJ/mol). The self-assembly of the four SAMuls in Figure 7a was also monitored by CD (above the respective CMC), which indicated that the nanoassemblies had equal and opposite chirality. However, DLS measurements suggested that the diastereoisomers formed slightly different spherical micelles, the ll and dd assembling into smaller, better defined aggregates with lower surface charge (D_mic_ = 6.5 ± 2.9 and 7.2 ± 2.2 nm and ζ = 35.5 ± 3.3, 39.2 ± 2.2 mV) with respect to dl/ld (D_mic_ = 9.2 ± 2.8 and 8.6 ± 1.6 nm and ζ = 46.8 ± 0.5, and 43.3 ± 0.6 mV).

**Figure 8:**
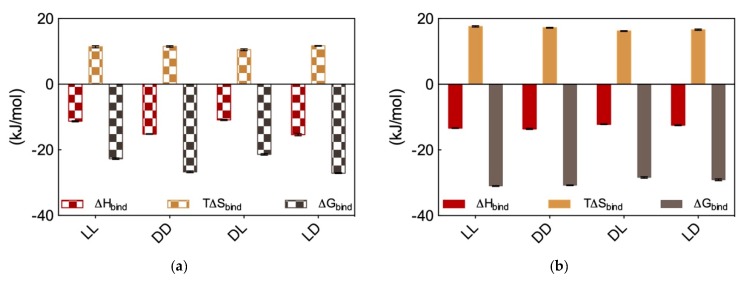
ITC-derived thermodynamic data for the binding of the micelles generated by the four chiral SAMuls C_16_-l-Ala-l-Lys (ll), C_16_-d-Ala-d-Lys (dd), C_16_-d-Ala-l-Lys (dl) and C_16_-l-Ala-d-lys (ld) to (**a**) DNA and (**b**) heparin. Adapted from [8], published by John Wiley & Sons, 2018.

In the light of these somewhat unexpected results, we proceeded with performing DNA and heparin binding via ITC. These results are reported in Figure 8a and Figure 8b, respectively. Considering DNA binding first, data in Figure 8a show that the dd and ld micelles displayed higher DNA affinity (∆G_bind_ = −26.7 ± 0.3 and −27.1 ± 0.1 kJ/mol, respectively) with respect to those formed by the alternative couple ll and dl (∆G_bind_ = −22.7 ± 0.2 and −21.4 ± 0.1 kJ/mol, respectively). Overall, the affinity for DNA of these SAMul micelles decreases in the order ld > dd > ll > dl. Importantly, the difference in ∆G_bind_ between the DNA best binder (ld) and the micelles presenting the lowest affinity for this polyanion (dl) is large (5.7 kJ/mol), and substantially larger than that measured for the related systems discussed above (Section 2.3), for which this difference amount to 2.6 kJ/mol. Also, further analysis of the thermodynamic data in Figure 8a reveals that enthalpy is governing the interaction between these four micelle types and DNA (∆H_bind_ = −15.4 ± 0.3, −15.2 ± 0.1, −11.3 ± 0.2, and −10.9 ± 0.2 for ld, dd, ll, and dl, respectively) although also T∆S_bind_ affords a favorable (i.e., positive) yet almost constant contribution to the interaction (T∆S_bind_ = +11.7 ± 0.1, +11.5 ± 0.2, +11.4 ± 0.3, and +10.5 ± 0.3 for ld, dd, ll, and dl, respectively). This last result is in line with the shape persistent nature of DNA discussed in Section 2.1, according to which this polyanion is better able to optimize interactions for enthalpic gain by virtue of its well-organized and repetitive structure along its double helix.

Moving on to heparin binding, from the ITC data reported in Figure 8b it appears immediately that not only all these four chiral SAMul micelles are able to bind the polysaccharide better than DNA but also, and perhaps more importantly, they show substantially less polyanion chiral recognition. Quantitatively, the ∆G_bind_ values for heparin binding by these nanomicelles are the following: −31.1 ± 0.1, −30.8 ± 0.1, −28.4 ± 0.2, and −29.1 ± 0.3 kJ/mol for ll, dd; dl, and ld, respectively. Therefore (although in this case with a considerably lower difference between the “stronger” and “weaker” binder then in the case of DNA), the affinity for the polysaccharide by these SAMuls follows the order: ll ≥ dd > ld ≥ dl. As it is also evident from data in Figure 8b, and contrarily to what observed for DNA, heparin binding is entropically driven (T∆S_bind_ = +17.6 ± 0.2, +17.2 ± 0.1, +16.2 ± 0.1, and +16.6 ± 0.2 kJ/mol for LL, DD; DL, and LD, respectively), although also in this case enthalpy affords in all cases a favorable contribution (∆H_bind_ = −13.4 ± 0.1, −13.6 ± 0.2, −12.2 ± 0.1, and −12.5 ± 0.01 kJ/mol for ll, dd; dl, and ld, respectively). This is also in line with the adaptive character of the heparin polyanion (Section 2.1) which, with respect to the shape-persistent DNA, can re-organize its conformation to achieve a greater surface contact with the SAMul nanomicelles. This, in turn, leads to a more effective binding interface stabilized by–besides the expected electrostatic interactions (∆H_bind_ < 0)—hydrophobic contacts between the Lys CH_2_ groups and, above all, a substantial release of water molecules and counterions upon nanoscale binding interface formation (T∆S_bind_ > 0).

In aggregate, these results are quite important, as they led us to formulate two major concepts: (i) with respect to Gly, the presence of the chiral spacer Ala in the inner part of the SAMul ligand head assists the terminal chiral Lys residue in its pre-organization for DNA enantiomeric recognition, and (ii) while the stereochemical configuration of the inner spacer in the SAMul ligand head does not play a role in DNA recognition, that at the chiral center of the terminal binding unit does, as micelles featuring SAMul the terminal binding unit in the D configuration (e.g., ld or dd) are considerably more affine to DNA then those having the surface Lys with opposite chirality (ll or dl). On the contrary, the ability of adaptive, ill-defined heparin binding is primarily controlled by the ability of the SAMul systems to self-assemble irrespective of the chirality presented at the binding interface, with heparin wrapping around the nanosystems but not forming highly optimized electrostatic interaction with the chiral nanomicelles.

## 3. Conclusions

In this work we presented our own experience in uncovering new concepts in the binding process of two fundamental biological polyanions—DNA and heparin—by nanomicelles formed by different SAMul amphiphilic ligands. These studies allowed us to highlight new concepts and some misconceptions in the field, summarized as follows: (i) fundamental differences exist between the binding properties of the two polyanions, suggesting that DNA is a shape-persistent binder while heparin in endowed with a more adaptive character; (ii) charge density is not the only main player in the electrostatic binding between the positively charged SAMul micelles and the negatively charged polyanions, as selective polyanion recognition strongly depends on the precisely defined details of each SAMul architecture; (iii) chiral SAMul molecules can exert selective polyanion binding when assisted by specific inner molecular features, which orchestrate the pre-organization of the chiral terminal groups in the formation of productive binding interfaces. Table 3 summarizes and highlight the differential affinity of all SAMul micelles towards the two polyanions with respect to the underlying investigated effect.

Aside from the fundamental valency of these studies, the results discussed in this review can have a practical impact on a range of biological/biomedical applications, including (but not limited to) drug and gene delivery—where the design of optimized SAMul-based systems could be employed for effective drug/DNA/RNA delivery nanovectors—and coagulation control during major surgery operation in which, e.g., clotting time could be controlled using SAMul nanomicelles in place of e.g., protamine, as heparin modulators.

## Figures and Tables

**Table 1:** Critical micelle concentration (CMC), thermodynamic data of micellization (free energy of micellization ∆G_mic_, micellization enthalpy ∆H_mic_, and micellization entropy T∆S_mic_), average micellar diameters (D_mic_), aggregation number (N_agg_), surface charge density (σ_mic_), and zeta potential (ζ) for the micelles formed by the SAMul ligands C_16_-DAPMA, C_16_-SPD, ad C_16_-SPM in buffered solution at physiological pH and ionic strength (7.4, 150 mM NaCl). CMC, ∆G_mic_, ∆H_mic_, and T∆S_mic_ values were obtained from Isothermal Titration Calorimetry (ITC) experiments. D_mic_ and ζ values were estimated by Dynamic Light Scattering (DLS) measurements, while N_agg_ and σ_mic_ values were predicted from MD simulations. Adapted from [13], published by RSC, 2016.

	C_16_-DAPMA	C_16_-SPD	C_16_-SPM
CMC (μM)	34	52	71
∆G_mic_ (kJ/mol)	−25.52	−24.47	−23.70
∆H_mic_ (kJ/mol)	−10.81	−8.61	−8.41
T∆S_mic_ (kJ/mol)	14.72	15.86	15.29
D_mic_ (nm)	6.2 ± 1.3	6.6 ± 0.2	6.2 ± 0.1
N_agg_ (-)	16 ± 2	13 ± 1	10 ± 1
σ_mic_ (e/nm^2^)	0.026	0.024	0.026
ζ (mV)	+51.9 ± 2.6	+44.0 ± 1.7	+40.5 ± 0.9

**Table 2:** Experimental and computational (in parenthesis) characterization of the self-assembled micelles formed by the three SAMul ligands featuring different degrees of unsaturation in their hydrophobic part in 150 mM NaCl buffered solution (10 mM Tris-HCl, pH = 7.4). The aggregation number N_agg_ was derived from MD simulations. Adapted from [12], published by RSC, 2017.

SAMul Ligand	D_mic_ (nm)	ζ_exp_ (mV)	CMC (μM)	N_agg_ (-)
C_18,1_-DAPMA_0_	5.2 ± 0.5 (5.4 ± 0.4)	+64.1 ± 0.6 (+63)	42 ± 3	28 ± 2
C_18,2_-DAPMA	6.4 ± 0.4 (6.2 ± 0.2)	+72.9 ± 3.7 (+73.4)	82 ± 2	32 ± 1
C_18,3_-DAPMA_0_	7.6 ± 0.3 (7.2 ± 0.2)	+72.9 ± 2.5 (+75.2)	78 ± 10	35 ± 1

**Table 3:** Summary of the differential affinity of all SAMul micelles towards heparin and DNA with respect to the underlying investigated effect.

Effect	SAMul Ligand	DNA Affinity	Heparin Affinity
Surface groups in polyanion recognition	C_16_-DAPMA	+ +	+
	C16-SPD	+ +	+ + +
	C16-SPM	+ + +	++
Hydrophobic tails in polyanion recognition	C_18,1_-DAPMA	+	+ + +
	C_18,2_-DAPMA	+ +	+ +
	C_18,3_-DAPMA	+ + +	+
Chirality in polyanion recognition	C_16_-l-Lys	+ +	+ + +
	C_16_-d-Lys	+ +	+ + +
	C_16_-Gly-l-Lys	+	+
	C_16_-Gly-d-Lys	+ + +	+ +
Chirality in polyanion recognition—revisited	C_16_-l-Ala-l-Lys	+	+ + +
	C_16_-d-Ala-d-Lys	+ + +	+ + +
	C_16_-d-Ala-l-Lys	+	+ +
	C_16_-l-Ala-d-Lys	+ + +	+ +
